# Association between glucagon-like peptide-1 receptor agonists use and change in alcohol consumption: a systematic review

**DOI:** 10.1016/j.eclinm.2024.102920

**Published:** 2024-11-14

**Authors:** Mohsan Subhani, Ashwin Dhanda, James A. King, Fiona C. Warren, Siobhan Creanor, Melanie J. Davies, Sally Eldeghaidy, Stephen Bawden, Penny A. Gowland, Ramon Bataller, Justin Greenwood, Stephen Kaar, Neeraj Bhala, Guruprasad P. Aithal

**Affiliations:** a Nottingham Digestive Diseases Centre (NDDC), Translational Medical Sciences, School of Medicine, University of Nottingham, NG7 2UH, UK; b NIHR Nottingham Biomedical Research Centre, Nottingham University Hospitals NHS Trust and the University of Nottingham, Nottingham NG7 2UH, UK; c Faculty of Health, University of Plymouth, Plymouth PL4 8AA, UK; d National Centre for Sport and Exercise Medicine, School of Sport, Exercise and Health Sciences, Loughborough University, Loughborough LE11 3TU, UK; e NIHR Leicester Biomedical Research Centre, University Hospitals of Leicester NHS Trust and the University of Leicester, UK; f Faculty of Health and Life Sciences, University of Exeter, Exeter EX4 4QJ, UK; g Diabetes Research Centre, University of Leicester and the National Institute for Health Research Leicester Biomedical Research Centre, Leicester General Hospital, Leicester LE5 4PW, UK; h Sir Peter Mansfield Imaging Centre School of Physics and Astronomy, University of Nottingham, University Park, Nottingham NG7 2UH, UK; i Division of Food, Nutrition & Dietetics, School of Biosciences, University of Nottingham, Loughborough LE12 5RD, UK; j Liver Unit, Hospital Clinic, Barcelona, Spain; k Division of Psychology and Mental Health, School of Health Sciences, University of Manchester, M139WL, UK; l Institut d’Investigacions Biomèdiques August Pi i Sunyer (IDIBAPS), Barcelona, Spain

**Keywords:** Glucagon-like peptide-1 receptor agonists, GLP-1 agonist, Alcohol use, Alcoholism, Systematic review

## Abstract

**Background:**

Despite the availability of various pharmacological and behavioural interventions, alcohol-related mortality is rising. This systematic review aimed to critically evaluate the existing literature on the association between glucagon-like peptide-1 receptor agonists use (GLP-1 RAs) and alcohol consumption.

**Methods:**

Electronic searches were conducted on Ovid Medline, EMBASE, PsycINFO, clintrials.gov, and ProQuest until the end of March 2024. An updated search was done on 7th of August 2024. The primary outcome was to explore the association between GLP-1 RAs use and change in alcohol consumption. Secondary outcomes included evaluating the impact of GLP-1 RAs on occurrences of alcohol-related events, healthcare utilisation, and the effect on functional magnetic resonance imaging (fMRI) cue reactivity. This study is registered with PROSPERO #CRD42024531982.

**Findings:**

Six studies totalling 88,190 participants were included with 38,740 (43.9%) receiving GLP-1 RA, but only 286 participated in randomised controlled trials. Pooled mean age was 49.6 years (SD = 10.5). RCT data did not show a reduction in alcohol consumption over 30 days after 24 weeks of treatment with exenatide versus placebo (heavy drinking days 6.0 [higher in control group], 95% CI −7.4 to 19.4, p = 0.37), a subgroup analysis found a positive effect in people with obesity (BMI >30 kg/m^2^), with significant reductions in brain reward centre cue reactivity on fMRI. In a secondary analysis of an RCT participants taking dulaglutide compared to placebo were 29% more likely to reduce alcohol intake (relative effect size 0.71, 95% CI 0.52–0.97, p = 0.04). Observational studies showed fewer alcohol-related healthcare events and a significant reduction in alcohol use with GLP-1 RAs treatment compared to DPP4-Dipeptidyl peptidase 4 use, no treatment and/or alcohol intake at baseline.

**Interpretation:**

There is little high-quality evidence demonstrating the effect of GLP-1 RAs on alcohol use. Subgroup analysis from two RCTs and supporting data from four observational studies suggest that GLP-1 RAs may reduce alcohol consumption and improve outcomes in some individuals. Heterogeneous study findings warrant further research to establish the effectiveness and safety of GLP-1 RAs in this population.

**Funding:**

10.13039/501100000272National Institute for Health and Care Research (NIHR): Award-ID: NIHR155469; NIHR154191; NIHR155530. 10.13039/501100020624NIHR Nottingham Biomedical Research Centre, Award-ID: BRC-1215-20003.

**Research in context**

**Evidence before this study**

Despite various available interventions, alcohol-related harm continues to rise. In 2024, the United Kingdom (UK) reported its highest number of alcohol-specific deaths ever recorded, with over 10,000 people dying from alcohol-specific causes every year. Obesity rates are also rising, affecting over 25% of adults and up to 50% of those with alcohol-related liver disease (ARLD). Currently, no interventions target both obesity and alcohol use disorder (AUD) simultaneously a new entity known as metabolic and alcohol-associated liver disease (MetALD). We systematically evaluated the existing literature on glucagon-like peptide-1 receptor agonists (GLP-1 RAs) use and change in alcohol consumption. We electronically searched Ovid Medline, EMBASE, and PsycINFO from inception to August 2024 to identify studies exploring the role of GLP1RAs in people with excess alcohol intake. We employed search terms, including controlled vocabulary, to identify keywords related to each component of the research question. The assessment of study quality and risk of bias for the included studies was conducted utilizing the Critical Appraisal Skills Programme (CASP) tool.

**Added value of this study**

In this systematic review, we evaluated 6 articles including 2 randomised control trials comprising 88,190 participants. Of these, 38,740 (43.9%) participants received GLP-1 RA. Our finding shows that GLP-1 RAs, initially developed for type 2 diabetes and obesity, have shown some promise in reducing alcohol consumption, potentially by targeting the brain’s reward centre, especially in people with body mass index ≥ 30 kg/m^2^. Moreover, the observational data shows the use of GLP1 RA was associated with a significant reduction in alcohol-associated events. The safety profile of GLP1RAs, in this population, was similar to previously reported data with mild gastrointestinal symptoms commonly reported adverse events.

**Implications of all the available evidence**

The potential use of GLP-1 RAs to reduce alcohol intake through their action at the brain reward centre opens novel therapeutic options to mitigate rising alcohol-related mortality and multimorbidity. The finding indicated the need for more definitive research to prove the effectiveness of GLP-1 RAs in reducing alcohol consumption and explore the underlying mechanisms.

## Introduction

Excessive alcohol drinking remains a significant global health concern, contributing to a wide range of social, economic, and health-related problems.^1^ In the United Kingdom (UK), 25% of the population regularly consumes alcohol in an amount that exceeds clinical recommendations. Moreover, data indicate that alcohol-related disorders (ARD) are among the most common reasons for hospitalisation in England, with UK-wide alcohol-related deaths peaking in 2022 (n = 10,048). Whilst ARD costs the National Health Service over £3.5 billion per annum the wider costs to society are even greater (£21 billion).^2^

Excessive drinking may lead to alcohol use disorder (AUD), characterised by a loss of control over alcohol consumption despite the adverse consequences.^3^ AUD is mediated by pathological changes in brain neurobiology relating to motivated behaviour, emotion and stress management. Dysregulated neurotransmitter systems, particularly the mesolimbic dopamine pathway, are prominent; however, the reinforcing effects of alcohol are also influenced by serotonin, opioid peptides, gamma-aminobutyric acid, and glutamatergic systems.^4^ Existing pharmacotherapies for excess alcohol consumption primarily target these pathways but their efficacy is often limited by adverse effects and poor adherence.^5^

Although licensed as therapies for type 2 diabetes and obesity, interest has been gathered concerning the potential of glucagon-like peptide-1 receptor agonists (GLP-1 RAs) for AUD. GLP-1 is an incretin hormone involved in glycaemic control and appetite regulation. Its potential in AUD is related to possible interactions with central pathways implicated in craving, reward and addiction, including alcohol dependence.^6^ Specifically, GLP-1 receptors are widely distributed throughout the cerebral cortex, hypothalamus, hippocampus, thalamus, caudate nucleus and globus pallidus.^7^ Importantly, GLP-1 receptors are widely expressed in the mesolimbic reward pathway which is central to the neurobiology of addiction.^7^

Preclinical studies have provided compelling evidence supporting the potential of GLP-1 RA in reducing alcohol consumption and reinforcing change in high-risk drinking behaviour.^6^^,^^8^^,^^9^ Animal models of AUD have demonstrated that GLP-1 RAs attenuates alcohol-seeking behaviour, decreases alcohol intake, and mitigates withdrawal symptoms through modulation of neurotransmitter release and neuronal activity within the mesolimbic circuitry.^10^^,^^11^ These findings have spurred clinical investigations to evaluate the use of GLP-1 RAs in people with AUD.

Wider evidence supports the potential of GLP-1 RAs for the treatment of reward system-related disorders. For instance, based on preclinical data, a recent systematic review reported that GLP-1 RA also decreased addictive behaviour relating to cocaine and nicotine, with less consistent evidence for opioids.^12^ A number of recent clinical investigations have attempted to replicate these findings; however, trials are sparse, heterogeneous, and limited by small sample sizes. Whilst some studies are ongoing, published clinical data demonstrate positive effects of GLP-1 RA on binge eating disorder, no effect on cocaine use disorder, and mixed effects on smoking cessation.^13^, ^14^, ^15^, ^16^

Beyond direct effects on alcohol consumption, GLP-1 RAs benefit obesity and type 2 diabetes; collectively improving outcomes for those whose liver disease has both metabolic and alcohol-related causes (MetALD).^17^ However, a comprehensive assessment of their long-term efficacy, safety, and tolerability in patients with excess alcohol consumption and AUD is necessary. As a first step, this systematic review sought to synthesise existing evidence regarding the interaction between GLP-1 RAs and alcohol intake in people who consume alcohol excessively. A secondary aim of our review was to map existing literature regarding study design, characteristics of participants, interventions, and research outcomes.

## Methods

This systematic review was conducted and reported in accordance with PRISMA guidelines for systematic reviews.^18^ This systematic review involved the collection and analysis of data from previously published studies and did not involve any direct participation of human or animal subjects. Since systematic reviews involve synthesising existing data from published studies no ethical approval or individual participant consent was required.

An electronic search was conducted on the 24th of March 2024 using Ovid Medline, EMBASE, and PsycINFO to identify articles published from inception to the date of search. Additionally, references of retrieved articles were manually screened, and grey literature was sought through clintrials.gov, Google Scholar, and ProQuest. An updated literature search was conducted on August 7, 2024, using revised terms (Supplementary Table S1) and no additional papers were added. The search strategy followed a Population, Intervention, Comparator, and Outcome (PICO) model, with input from a local expert librarian for strategy refinement. The review was registered with PROSPERO (CRD42024531982).

The inclusion criteria for individual studies and indications for GLP-1 RAs use are provided in the Supplementary material (Supplementary Table S2).

### Search strategy

Different combinations of the following search terms were used:

“Glucagon-Like Peptide 1” or “glp1 or “glp-1” or “glucagon like peptide 1” or “glucagon like peptide one” or “Glucagon-Like Peptide-1 Receptor” or “semaglutide” or “exenatide” or “liraglutide” or “dulaglutide” or “Tirzepatide” or “Lixisendatide” or “Albiglutide” AND “Alcoholism” or “Alcohol-Related Disorders” or “alcoholism” or “alcohol use disorder∗” or “AUD” or “high risk drinking behaviour” or “high risk drinking behavior” or “binge drink∗” or “problem drinking” or “ARLD” or “MetALD” or “ethanol abuse” or “alcohol related disorder∗” or “alcohol associated liver disease∗” or “alcohol related liver disease∗”

The sample search strategy for Ovid Medline, Embase, and PsycINFO is given in supplementary material (Supplementary Table S1).

### Eligibility

We included studies involving people with occasional or excess alcohol consumption including but not limited to AUD, of any research design, reporting on: 1) associations between GLP-1 RA and alcohol-related outcomes; and 2) effects of GLP-1 RA on alcohol-related outcomes. Manuscripts reporting reviews and pre-clinical data were not included. Peer-reviewed journal articles were included if they were written in English and involved human participants. Published abstracts were considered for inclusion and underwent the same quality assessment procedures as full-text articles. On-going clinical trials were included if trial records provided the necessary detail to judge eligibility. Excess alcohol consumption diagnosis criteria included alcohol consumption >14 units per week, a physician’s diagnosis, or identification via validated assessments such as the Alcohol Use Disorders Identification Test (AUDIT) score, International Classification of Diseases 10 (ICD 10)^19^ or Diagnostic and Statistical Manual of Mental Disorders 5 (DSM 5) Criteria.^20^ In addition, we included studies which reported the impact of GLP 1 RAs on occasional or any alcohol use.

### Screening and data extraction

**Fig. 1:**
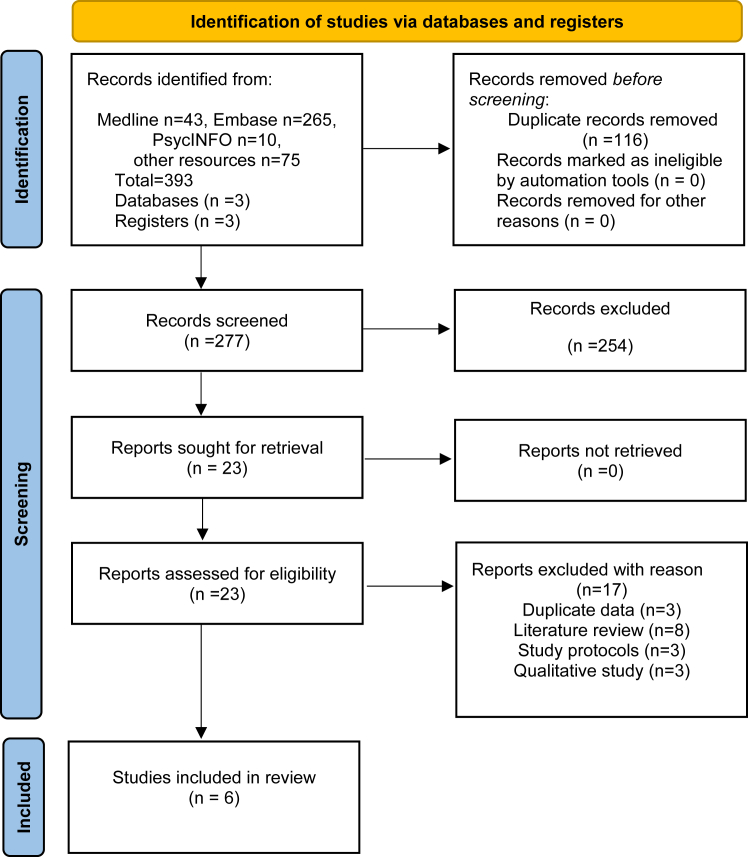
PRISMA 2020 flow diagram for study inclusion.

After the removal of duplicates, two reviewers (MS and JK) independently screened the titles and abstracts for eligibility and recorded decisions using Rayyan-QRCI systematic review software, Endnote (version-X9) and Microsoft Excel. A third reviewer (AD) oversaw the process and resolved any conflicts in discussion with the senior author (GPA). Reasons for the exclusion of ineligible studies were recorded and the selection process was recorded in a flow diagram (Fig. 1). A tailored data extraction form was developed with the Cochrane checklist as a reference. Two reviewers (MS and JK) extracted relevant data using a standard template for data extraction. Subsequently, a third reviewer (AD) cross-checked the data for accuracy and consistency. In cases of missing data, abstract-only publication and data enquiry, the corresponding author of the study was contacted.

### Outcomes and results synthesis

The primary outcome was to explore the impact of GLP-1 RA on self-reported alcohol intake in people who drink alcohol. Secondary outcomes included evaluating the impact of GLP-1 RAs on healthcare utilisation and occurrences of alcohol-related health events. Additional outcomes included occurrences of adverse events, and the effects on functional magnetic resonance imaging (fMRI) cue reactivity.

For included studies, we abstracted data on research location (country), study design, publication year, author names, participant characteristics, sample size, inclusion criteria (including AUD diagnoses), and alcohol intake measures. Adverse and serious adverse events were also extracted from clinical trials.

Extracted data were organised and synthesised to most clearly communicate the key findings regarding our primary and secondary outcomes. Therefore, evidence surrounding the interaction between GLP-1 RA and drinking behaviour is presented first, broken down by study design (clinical trials versus observational studies). Evidence relating to secondary outcomes are then summarised in tabular format (Table 1) (i.e. study design, interventions, research outcomes, characteristics of participants, adverse events). Details of fMRI outcomes are reported narratively. Primary and secondary outcomes are summarised in tabular form (Table 2).

**Table 1:** Characteristics of included studies and population.

Author (year)	Country setting	Design	Intervention	Control	Duration	Follow-up	Population						Baseline self-reported alcohol measures	
							Inclusion criteria	Size (male)	Ethnicity	Age (Years)	Intervention (male)	Control (male)	Intervention	Control
Klausen et al. (2022)	Denmark Copenhagen out-patient clinics	RCT single centre	Exenatide	Placebo	26 weeks (treatment)	6 months	Age 18–70 years DSM-5 AUD or ICD-10 alcohol dependence, and treatment seeking	Recruited 127 (76), FU at 26 weeks = 58, FU at 6 months = 55	White Danish (127)	Mean = 52.0 (SD = 10.0)	62 (37)	65 (39)	ICD-10 alcohol dependence = 62; DSM-5 AUD: mild = 7, moderate = 5, severe = 50; heavy drinking days: ≤17 = 35 and 18–30 = 27	ICD-10 alcohol dependence = 65; DSM-5 AUD: mild = 4, moderate = 7, severe = 54; heavy drinking days: ≤17 = 35 and 18–30 = 30
Kalra et al. (2024)^a^	India, Endocrine clinic	Retrospective, observational	Liraglutide			3 months	Started on liraglutide from the endocrine clinic	69 (69), 42 admitted to alcohol intake	Indian		42		MAST score: >5 = 14, mean = 3.0 (SD = 3.5); consuming: ≥200 mL/day = 6, ≥30 mL/day = 21	
Probst et al. (2023)	Switzerland, University Hospital Basel	Secondary analysis of RCT Single centre	Dulaglutide	Placebo	12 weeks (treatment)	12 weeks	Age 18–75 years, smoker, willing to stop smoking	total 255, AUD 159 (62), AUD data at 12 weeks 151 (59)	White = 157, African = 1, Hispanic = 1	Median = 42.0 (IQR 33–53)	76 (25)	75 (34)	Heavy drinker^b^ = 10	Heavy drinker = 8
Quddos et al. (2023)	USA, Community	Prospective, Cohort control	Semaglutide or Tirzepatide	None	Timeline follow back for last 30 days	30 days	≥21 years, BMI ≥ 30, on Semaglutide or Tirzepatide, current alcohol drinker	153 (29)	White = 136, Asian = 1, Black = 9, Native American = 1, other = 6	Control 38.9 (SD = 11.3), semaglutide 41.6 (SD = 9.1), tirzepatide 43.7 (SD = 8.2),	Semaglutide = 56 (9), tirzepatide = 50 (9)	47 (11)		
Wium-Andersen et al. (2022)	Denmark, nationwide database	Retrospective, observational	Any GLP1 analogue	DPP4	Recruitment: 2009–2017, Alcohol-related events 2009–2018	4.1 years	Anyone with a new prescription for GLP1 or DPP4 during the study period^c^	87,676 (49,942)		GLP-1 57.8 (12.1), DPP4 65.1 (12.5)	GLP-1 = 38,454 (21,381), DPP4 = 49,222 (28,562)		Alcohol or substance abuse: GLP-1 = 2,040, DPP4 = 2602	
Richards et al. (2023)	USA	Retrospective, Case series	Semaglutide or Tirzepatide	None			Adult, on semaglutide, AUD	6	White = 6					

AUD-alcohol use disorder, AUDIT-alcohol use disorder identification test, BMI-Body mass index, DPP4-Dipeptidyl peptidase 4, FU-follow-up, GLP-1- glucagon-like peptide 1, MAST-Michigan alcohol screening test, RCT-randomised control trial, SD = standard deviation, SE−standard error.

a Conference abstract.

b Female participants drinking more than 7 and male participants drinking more than 14 glasses of alcohol per week.

c (1) Hospital contacts with a main diagnosis of alcohol use disorders (international classification of diseases [ICD]-10 code DF10) in the Danish National Patient Registry, (2) registered treatments for alcoholism in the National Registry of Alcohol Treatment or (3) purchase of the benzodiazepine chlordiazepoxide registered in the Danish National Prescription Registry.

**Table 2:** Change in self-reported alcohol intake measures.

Author (year)	Change in self-reported alcohol measures
Klausen et al. (2022)	**Exenatide = At 26 Weeks** Heavy drinking days: −19.6 (95% CI –27.4 to −11.8); AUDIT: −7.0 (95% CI –8.8 to −5.1); PACS: −5.4 (95% CI –7.0 to −3.9). **At 6 months** Heavy drinking days: −3.2 (95% CI–5.9 to −0.5). **Placebo = At 26 Weeks** Heavy drinking days: −26.8 (95% CI −34.4 to −19.2), p = 0.11; AUDIT: −8.2 (95% CI −10.0 to −6.5), p = 0.59; PACS: −7.3 (95% CI −8.8 to −5.8), p = 0.42. At 6 months Heavy drinking days: −5.6 (95% CI −8.4 to −2.7), p = 0.18
Kalra et al. (2024)^a^	Abstinence = 9; reduce drinking = 33; MAST score: >5 = 4, mean = 2.0 (SD = 1.8) p < 0.05
Probst et al. (2023)	Relative effect size for a reduction in alcohol intake compared to placebo = 0.71 (95% CI 0.52–0.97, p = 0.04); Relative effect size for a reduction in alcohol intake compared to placebo (adjusted for education):0.64 (95% CI 0.47–0.86, P = 0.004)
Quddos et al. (2023)	Change compared to control: number of drinks- Sem: B = −1.31 (SE = 0.3, p ≤ 0.001), Tirzepatide = −1.54 (SE = 0.31, p ≤ 0.001); binge drinking- Sem: B = −2.05 (SE = 0.6, <0.001), Tirzepatide: B = −3.8 (SE = 0.68, p ≤ 0.001); AUDIT- Sem: B = −5.1 (SE = 1.3, p < 0.001), Tirzepatide: B = −6.7 (SE = 1.3, p < 0.001)
Wium-Andersen et al. (2022)	Alcohol-related episodes GLP-1 compared with the use of DPP-4: ITT- adjusted HR- 0–90 days: 0.46 (SD = 0.24–0.86), 90–365 days: 0.98 (SD = 0.64–1.49), 0–365 days: 0.76 (SD = 0.53–1.07), 356–4619 days: 0.72 (SD = 0.60–0.86)
Richards et al. (2023)	The mean reduction in AUDIT: 9.5 points, p < 0.001; reduction in alcohol intake = 6

a Conference abstract publication.

The assessment of study quality and risk of bias for the included studies was conducted utilising the Critical Appraisal Skills Programme (CASP) tool.^21^ Studies were categorised into three quality levels—low, medium, or high—based on their responses to the assessment questions. Studies providing satisfactory information across all domains were deemed high quality. Those with missing or unsatisfactory information in one domain were considered medium quality. Studies lacking adequate information in two or more domains were classified as low quality.

### Role of the funding source

The funder of the study had no role in study design, data collection, data analysis, data interpretation, or writing of the report.

## Results

A total of 1128 records were initially identified. After removing duplicates and applying eligibility criteria, six studies were included in the final narrative synthesis^22^, ^23^, ^24^, ^25^, ^26^, ^27^ (Fig. 1). Of the included studies, two were randomised controlled trials,^22^^,^^23^ one case series,^25^ and three retrospective observational studies.^24^^,^^26^^,^^27^ Three studies were from Europe,^22^^,^^23^^,^^27^ two from the United States^24^^,^^25^ and one from India.^26^ The characteristics of the included studies are summarised in Table 1.

### Participants

A total of 88,190 participants (RCT n = 286, observational studies n = 87,904) were included. The pooled mean age was 49.6 (SD = 10.5) years), 56.9% (50,184/88,190) were male, and in five studies where ethnicity was provided 82.9% (426/514) were white. In two RCTs, 138 (48.3%) participants received GLP-1 RA (exenatide n = 62, dulaglutide n = 76) and 148 (51.7%) received placebo. In observational studies, one study had no control arm and all 42 participants received liraglutide.^26^ In the study by Quddos et al. (2023), 56 participants were on semaglutide, 50 on tirzepatide and 47 in the control arm did not receive any drug.^24^ Most participants were from a single retrospective observational cohort study of 87,676 individuals, of whom 38,454 received a GLP-1 RA and 49,222 dipeptidyl peptidase 4 (DPP4).^27^ The baseline self-reported alcohol measures and body mass index (BMI) are given in Table 1.

### Interaction between GLP-1 RA and self-reported alcohol measures

In a randomised controlled trial of exenatide versus placebo in 127 participants with AUD, assessed as high quality, Klausen et al. (2022), reported no significant difference in self-reported alcohol measures after 26 weeks of treatment (estimated treatment difference (exenatide versus placebo; positive values favour control and negative favour exenatide): heavy drinking days 6.0, 95% CI −7.4 to 19.4, p = 0.37; total alcohol consumption g/30 days −42.0, 95% CI −507.7 to 423.7, p = 0.86; days without alcohol use −10.5, 95% CI −2.6 to 23.4, p = 0.11; and AUDIT 1.1, 95% CI −2.9 to 5.0, p = 0.59), or at 6 months after completion of treatment (estimated treatment difference: heavy drinking days 2.6, 95% CI −1.3 to 6.5, p = 0.93).^22^

In a pre-defined secondary analysis of a randomized controlled trial of dulaglutide versus placebo for smoking cessation, assessed as high quality, 129 of the 255 participants consumed alcohol.^23^ After 12 weeks of treatment, participants who consumed alcohol at baseline and received dulaglutide drank 29% fewer glasses of alcohol per week compared to placebo (relative effect size 0.71, 95% CI 0.52–0.97, p = 0.04) (11). However, in the heavy drinkers (n = 18), there was no statistical difference in reduction in alcohol use between arms (p = 0.50).

In a prospective cohort study of 153 participants with obesity and alcohol use receiving either semaglutide or tirzepatide for at least 30 days, assessed as medium quality, Quddos et al. (2023) reported a significant reduction in the self-reported number of drinks (−1.31, SEM = 0.3 for semaglutide and −1.54, SEM = 0.31 for tirzepatide, both p ≤ 0.001) and binge drinking episodes (−2.05, SEM = 0.6 for semaglutide and −3.8, SEM = 0.68 for tirzepatide, both p ≤ 0.001) compared with controls (Table 1).^24^

In a retrospective uncontrolled observational study of 42 participants who received liraglutide, assessed as low quality, 21.4% (n = 9) reported abstinence and 78.6% (n = 33) reduced drinking (p < 0.05) after 3 months of treatment.^26^ The number of participants with a self-reported Michigan Alcohol Screening Test score greater than 5 (indicative of problem drinking) fell from 14 to 5 after 3 months (p < 0.05). In a retrospective case series of six patients with AUD who were prescribed semaglutide for weight loss, assessed as low quality, Richards et al. (2023) reported a mean reduction of 9.5 points in AUDIT score after 1–9 months of treatment.^25^ A summary of change in self-reported alcohol measures for individual studies is provided in Table 2.

### Association between changes in body mass index (BMI) and alcohol consumption

In an exploratory analysis, Klausen et al. (2022)^22^ demonstrated that in a subgroup of participants (n = 30) with BMI >30 kg/m^2^, exenatide compared with placebo induced a significant reduction in heavy drinking days (−23.6 percentage points, 95% CI, −44.4 to −2.7, P = 0.034) and total alcohol consumed (−1205 g, 95% CI, −2206 to −204, P = 0.026). Conversely, in patients with BMI < 25 kg/m^2^, exenatide increased heavy drinking days by 27.5 percentage points (95% CI, 4.7 to 50.2, P = 0.024) compared with placebo, with no significant difference in total alcohol intake.

### Association between GLP-1 RAs and alcohol-related health events/healthcare utilisation

An analysis of routinely collected healthcare data in Denmark (assessed as a medium-quality study) included all new users of GLP-1 RA (n = 38,454) and dipeptidyl peptidase-4 inhibitor (DPP4) (n = 49,222) between 2009 and 2017.^27^ During a median follow-up period of 4.1 (interquartile range [IQR] 2.1–6.9) years, 0.7% (649/87,676) participants experienced an alcohol-related event (incident rate 16.3 cases per 10,000 person-years, 95% CI 15.1–17.7). GLP-1 RA compared to DPP4 treatment was associated with fewer alcohol-related episodes (hospital contacts or treatment for AUD, used as a surrogate for self-reported alcohol use) in the first 3 months of treatment (Hazard ratio 0.46 (95%CI: 0.24–0.86). However, this effect was not present after longer durations of treatment.^27^

### Effect on functional brain imaging

Klausen et al. (2022), employed two functional MRI (fMRI) paradigms and single-photon emission CT (SPECT) to investigate CNS modulation by exenatide treatment in individuals with AUD.^22^ Utilising predefined regions of interest (ROIs), significant group effects on cue-reactivity were observed in the ventral striatum [F (1,31) = 4.744, P = 0.037, partial η2 = 0.133], dorsal striatum [F (1,31) = 6.124, P = 0.019, partial η2 = 0.165], and putamen [F (1,31) = 4.730, P = 0.037, partial η2 = 0.132] after 26 weeks of exenatide treatment compared with placebo. Notably, cue-induced activity was significantly reduced in the exenatide group compared with placebo (mean difference [M] = −0.176, SEM = 0.075, P = 0.025) in the ventral striatum at week 26. Exploratory whole-brain analysis revealed significant reductions in cue reactivity in the exenatide group compared to placebo, particularly in the caudate, septal area, and middle frontal gyrus. On the N-back fMRI task of working memory, there was an indication of an effect of group and SPECT imaging showed significantly lower dopamine transporter availability in the striatum in the exenatide group compared with placebo at week 26. These findings suggest a potential role for exenatide in modulating cue reactivity, working memory and the dopaminergic reward system in AUD.

On investigating fMRI subjective craving results, although initial analysis indicated a significant difference between the healthy controls and the patients at baseline (P < 0.001; mean (SD): healthy controls, 8.8 (15.96); placebo group, 33.5 (26.9); exenatide group, 30.6 (28.6). However, by the 26-week follow-up, this difference was no longer significant (P = 0.50; mean (SD): healthy controls, 8.8 (15.96); placebo group, 13.6 (12.0); exenatide group, 14.8 (23.07). The voxel-wise analysis of fMRI spatial working memory revealed a significant reduction in activation in the exenatide group at the week 26 rescan compared to the placebo group. This reduction was observed in response to the 2-back > 1-back task in two clusters located in the right frontal pole and right superior frontal gyrus, within the dorsolateral prefrontal cortex ROI. SPECT imaging showed significantly lower dopamine transporter availability in the exenatide group compared with placebo at week 26. These findings suggest a potential role for exenatide in modulating cue reactivity and neuronal activity associated with AUD.

### Adverse events reported in people receiving GLP-1 RA

In the Klausen et al. (2022) study, participants receiving exenatide experienced more gastrointestinal adverse events such as nausea, vomiting, and diarrhoea compared with the placebo group (47 events in 62 participants versus 23 in 65 participants, respectively).^22^ Probst et al. (2023), observed similar trends in gastrointestinal symptoms, though the incidence was higher.^23^ Other common adverse events reported were respiratory and urinary tract infections, injection site reactions, headaches and musculoskeletal symptoms. Hospitalisation due to alcohol withdrawal symptoms and depressive mood were the most reported adverse events.^22^^,^^23^ There was no data available on adverse events by Kalra et al. (2024), Quddos et al. (2023), Wium-Andersen et al. (2022), and Richards et al. (2023)^24^, ^25^, ^26^, ^27^ (Supplementary Table S3).

### Risk of bias assessment

Quality assessment stratified two studies as high quality, two as medium, and three as low quality. The main areas of concern were inconsistencies and errors in reported data (Table 3).

**Table 3:** Quality assessment of included studied.

Study ID	Study design		Study bias		Confounding		Results validity		Generalisability			
	Did the study address a clearly focused issue?	Was the cohort recruited in an acceptable way?	Was the exposure accurately measured to minimise bias?	Was the outcome accurately measured to minimise bias?	Have the authors identified all-important confounding factors?	Have they taken account of the confounding factors in the design and/or analysis?	How precise are the results?	Do you believe the results?	Can the results be applied to the local population?	Do the results of this study fit with other available evidence?	Does the study have implications for practice?	Overall quality
Klausen et al. (2022)	Yes	Yes	Yes	Yes	Yes	Yes	Highly Precise	Yes	Yes	Yes	Yes	High
Kalra et al. (2024)	Yes	Yes	Can’t tell	Can’t tell	Can’t tell	Can’t tell	Low Precise	Yes	Yes	Yes	Yes	Low
Probst et al. (2023)	Yes	Yes	Yes	Yes	Yes	Yes	Highly Precise	Yes	Yes	Yes	Yes	High
Quddos et al. (2023)	Yes	Yes	No	Can't tell	No	No	Moderately Precise	Yes	Yes	Yes	Yes	Low
Wium-Andersen et al. (2022)	Yes	Yes	Yes	Yes	No	Yes	Moderately Precise	Yes	Yes	Yes	Yes	Medium
Richards et al. (2023)	Yes	Yes	Yes	No	No	No	Low Precise	Yes	Yes	Yes	Yes	Low

The quality of included studies was assessed using the Critical Appraisal Skills Programme (CASP) 2018 checklist.

## Discussion

This systematic review identified a few studies investigating the interaction between GLP-1 RAs with alcohol consumption in people who drink excessively. This included two randomised controlled trials,^22^^,^^23^ of which only one was specifically designed to determine the efficacy of GLP-1 RAs for the treatment of AUD.^22^ Evidence from these trials did not demonstrate a consistent benefit of GLP-1 RAs in reducing alcohol use. Evidence from cohort and observational studies reported associations between GLP-1 RA treatment and reduction in alcohol use.^24^, ^25^, ^26^, ^27^ However, further robust evidence is required to determine whether GLP-1 RAs are an effective treatment for AUD.

The six eligible studies included in this systematic review tested and explored several GLP-1 RAs (liraglutide, semaglutide, dulaglutide, exenatide, and tirzepatide), which have differing clinical effectiveness for lowering glycaemia and weight.^28^ Studies encompassed various research designs, including randomised controlled trials and observational studies (case series and retrospective analyses). Across studies, diverse outcomes were reported, including changes in self-reported alcohol consumption, mechanistic investigations (functional brain imaging) and adverse events associated with GLP-1 RA use.

Only two high-quality studies, both RCTs, were included in this review.^22^^,^^23^ The Klausen study did not demonstrate a benefit of exenatide on alcohol use in people with AUD except in a sub-group of participants with obesity.^22^ The secondary analysis of an RCT reported by Probst (2023) demonstrated strong evidence for the effectiveness of a GLP-1 RA in reducing alcohol use in active alcohol drinkers with obesity - with a medium-to-large effect size to reduce alcohol use compared to a placebo after 12 weeks. However, this analysis did not find a difference in heavy drinkers.^23^ These contrasting results may be explained by the different GLP-1 RAs (exenatide and dulaglutide), duration of treatment (24 and 12 weeks) and baseline alcohol use. Both studies found a reduction in alcohol use in participants with obesity, while there was an increase in alcohol use in participants with BMI < 25 kg/m^2^ in the former.^22^ It should be noted that the secondary analyses in both studies included small numbers of participants (18 and 30) and were not sufficiently powered to detect an effect. While further mechanistic study is required to understand the differing effects of GLP-1 RAs in people with normal weight and obesity, functional deficits in GLP-1 signalling in people with obesity may be relevant.^29^

Two observational studies included participants taking tirzepatide,^24^^,^^25^ a dual agonist with activity for the GLP-1 and the glucose-dependent insulinotropic peptide (GIP) receptors. This drug has strong efficacy for the treatment of diabetes and obesity^30^^,^^31^ but there are no preclinical nor high-quality clinical studies investigating its effect on alcohol use. While these observational data suggest an association with reduced alcohol use in people with diabetes or obesity, it is unknown whether the powerful glucose-lowering effects of this drug may increase alcohol craving in people without these conditions.

Two qualitative studies providing a patient-centred perspective were identified but not eligible for inclusion in this review. In the first, a machine-learning mapping algorithm was applied to more than 68,000 social media posts relating to GLP-1 RAs on the Reddit platform.^32^ Of the 1580 posts relating to alcohol, 71% referred to a reduction in alcohol consumption, or a reduction in alcohol cravings and other alcohol-related negative events. A second qualitative study conducted a thematic analysis of online reports of alcohol use while taking GLP-1 RAs.^24^ The most prevalent themes were related to reduced alcohol use and craving. Abstinence was reported in approximately 10% of posts while about 3% did not notice a change in alcohol use. While these studies cannot demonstrate causation of effect, they do offer valuable insights into real-world patient experiences and perceptions regarding GLP-1 RA treatment and alcohol use.

This review identified several limitations in the existing literature. Heterogeneity in study designs, interventions, outcome measures, and patient populations, and lack of ethnic and gender diversity, hindered direct comparisons and generalisability of results. Only two studies were evaluated to be of high quality employing experimental designs. Additionally, variations in adverse event reporting across studies and incomplete data on safety profiles limited comprehensive risk assessment associated with GLP-1 RAs use.

The findings of this review align with previous literature indicating the potential of GLP-1 RAs in reducing alcohol consumption among individuals with AUD.^6^^,^^33^ A previous systematic review of the effect of GLP-1 RAs on substance use disorder identified 17 studies, all using rodent models, and demonstrated a benefit on the behavioural effects of alcohol, nicotine, amphetamine and cocaine.^6^ This highlights the strong underpinning preclinical evidence of the effect of GLP-1 RAs on alcohol and substance use disorders. Since this 2019 review, more efficacious GLP-1 RAs have been developed and tested in patient populations.

Functional and molecular brain imaging techniques have utility in providing mechanistic insights into the neurobiology and pathophysiology of alcohol use disorder.^34^ The findings in the septal area are particularly interesting as it is a region of the brain that is associated with reward^35^ and where GLP-1 receptors are highly expressed.^36^ Although there is growing evidence on the role of neuroimaging in AUD, at present studies on GLP-1 RA effects on brain regions implicated in addiction pathways, are limited to a single study.^22^ Moreover, further research is required to determine the optimal modality and timing of neuroimaging to distinguish the effect of intervention from those due to a reduction in alcohol consumption or abstinence.^37^ In addition, discrepancies in study outcomes and adverse event profiles underscore the need for further research to elucidate the efficacy and optimal use of GLP-1 RAs in reducing alcohol consumption in individuals with AUD, obesity, and liver disease. Additional studies are needed to determine the best GLP-1 RA dose and treatment duration.

There is a synergistic negative impact on the liver of alcohol and obesity, which increases the risk of advanced liver disease or death by 1.6-fold greater than the additive effect of each condition.^38^ With the high global prevalence of obesity and harmful alcohol use, MetALD is becoming increasingly common with up to 40% of people with AUD also living with obesity.^39^ GLP-1 RAs, if proven an effective treatment for excessive alcohol use, present a unique opportunity to treat both risk factors simultaneously. This may have a greater impact on liver disease outcomes than treating individual risk factors separately but will need to be evaluated in a high-quality RCT.

With this in mind, there is growing interest in this field with several randomised controlled trials underway (Table 4). An ongoing trial (NCT05895643) aims to assess whether semaglutide reduces alcohol intake in patients with AUD and comorbid obesity (BMI ≥ 30 kg/m^2^) over 26 weeks. Functional imaging techniques, including fMRI for alcohol cue reactivity and MRS for brain gamma-aminobutyric acid (GABA) levels, will be used. A terminated trial (NCT03645408) focused on exenatide’s effects on alcohol self-administration in heavy drinkers. A completed trial (NCT03232112) that is yet to report examined the impact of exenatide on alcohol intake in patients seeking treatment for alcohol dependence (10). Other ongoing trials (NCT05891587 and NCT06015893) are investigating the efficacy of semaglutide in reducing alcohol consumption and associated changes in brain activity using various functional neuroimaging techniques. Lastly, an ongoing study (NCT05892432) is exploring the effects of semaglutide on cue craving in adults with AUD. Study populations, interventions, and dosage protocols are unique to each trial. Findings from these trials will build on the existing evidence base by providing causative data on the effect of newer and more efficacious GLP-1 RAs on alcohol use in people with obesity and AUD and elucidating its central mechanisms of action.

**Table 4:** Ongoing randomised control trial investigating the role of glucagon-like peptide 1 receptor agonists in alcohol use.

NCT Number	Study title	Study status	Conditions	Interventions and control	Duration	Sample size	Sponsor	Inclusion criteria	Primary outcome measure	Functional imaging
NCT05895643	Does Semaglutide Reduce Alcohol Intake in Patients with Alcohol Use Disorder and Comorbid Obesity? Access online: https://clinicaltrials.gov/study/NCT05895643	RECRUITING	Alcohol use disorder	Semaglutide vs Placebo	26 weeks	108	Psychiatric Centre Rigshospitalet, Copenhagen, Denmark	Age 18–70 years, DSM-5 AUD or ICD-10 alcohol dependence, AUDIT score >15, BMI >30, heavy alcohol consumption in last >6/30 days	Change in heavy drinking days	fMRI alcohol cue-reactivity, MRS brain gamma-aminobutyric acid (GABA) levels
NCT03645408	The Effects of Exenatide, a GLP-1 Agonist, on Alcohol Self-Administration in Heavy Drinkers. Access online: https://clinicaltrials.gov/study/NCT03645408	TERMINATED	Alcohol Use Disorder	Exenatide vs Sham injection	Crossover study	28	Boston Medical Center, USA	Age 21–55 years, at least 1 episode of binge drinking per week, AUD, drink above the safe weekly limit	Alcohol consumption (effect exenatide on alcohol self-administration phase 1 trial)	None
NCT03232112	Does Treatment With GLP-1 Reduce Alcohol Intake in Patients with Alcohol Dependence? Access online: https://clinicaltrials.gov/study/NCT03232112	COMPLETED	Alcohol Dependence	Exenatide vs BD Posi Flush (saline)	26 weeks	127	Psychiatric Centre Rigshospitalet, Copenhagen, Denmark	Age 18–70 years DSM-5 AUD or ICD-10 alcohol dependence, and treatment seeking	Change in heavy drinking days	fMRI alcohol cue-reactivity
NCT05891587	Semaglutide Therapy for Alcohol Reduction - Tulsa. Access online: https://clinicaltrials.gov/study/NCT05891587	RECRUITING	Alcohol Use Disorder	Semaglutide vs: Placebo	12 weeks	80	Oklahoma State University Center for Health Sciences, USA	Age ≥ 18 years, AUD, CIWA ≤ 10, drinking more than the safe limit per week in the last 28 days	Difference in the number of standard alcoholic drinks consumed/week (Drinks Per Week	Changes in brain activity during an fMRI interoceptive attention task, during an alcohol-related Go/No-Go fMRI task and in response to alcohol cues during fMRI cue reactivity task
NCT06015893	Semaglutide Therapy for Alcohol Reduction (STAR). Access online: https://clinicaltrials.gov/study/NCT06015893	RECRUITING	Alcohol Use Disorder	Semaglutide vs Behavioural: Take Control	20 weeks	52	National Institute on Drug Abuse (NIDA), USA	Age ≥ 18 years, AUD, CIWA ≤10, drinking more than the safe limit per week in the last 28 days	Change in alcohol drinking amount and pattern. Safety and tolerability of semaglutide in AUD	Determine whether semaglutide reduces brain activity in resting-state and/or task-based fMRI scans.
NCT05892432	Clinical Trial of Rybelsus (Semaglutide) Among Adults with Alcohol Use Disorder (AUD). Access online: https://clinicaltrials.gov/study/NCT05892432	RECRUITING	Alcohol Use Disorder	Semaglutide 3 mg and Semaglutide 7 mg vs Placebo	8 weeks	135	University of Colorado, Denver, USA	Age ≥ 21 years, AUD, BMI ≥ 25	Change in Cue Craving Visual Analog Score	

In conclusion, this review underscores the promising yet heterogeneous evidence suggesting that GLP-1 RAs may reduce alcohol consumption and improve outcomes in some individuals. It highlights the need for further robust randomised controlled trials to determine GLP-1 RA efficacy, safety and cost-effectiveness in people with AUD.

## Contributors

Mohsan Subhani: Contributed to protocol writing, scoping search, drafting final Search Strategy, literature search, abstract screening, data extraction including critical appraisal, meta-analysis, report writing, and proofreading of the final manuscript. Ashwin Dhanda: Contributed to data extraction including critical appraisal, meta-analysis, report writing, and proofreading final manuscript. James A. King: Contributed to data extraction including critical appraisal, report writing and proofreading final manuscript. Fiona C Warren: Contributed to report writing and proofreading the final manuscript. Sioban E Creanor Contributed to report writing and proofreading the final manuscript Melanie J Davies: Contributed to finalising the research question, proofreading, and finalising the manuscript. Sally Eldeghaidy: Contributed to finalising the research question, proofreading, and finalising the manuscript. Stephen Bawden: Contributed to finalising the research question, proofreading, and finalising the manuscript. Penny A Gowland: Contributed to finalising the research question, proofreading, and finalising the manuscript. Ramon Bataller: Contributed to finalising the research question, proofreading, and finalising the manuscript. Justin Greenwood: PPIE co-author contributed to the research question, PPIE experience, proofreading, and finalising the manuscript. Stephen Kaar: Contributed to finalising the research question, proofreading, and finalising the manuscript. Neeraj Bhala: Contributed to finalising the research question, proofreading, and finalising the manuscript. Guruprasad Aithal: Senior Author: Contributed to finalising the research question, proofreading, developing the search strategy and protocol, statistical support, and finalising the manuscript. He also acted as 3rd reviewer in case of disagreement among primary reviewers.

All authors approved the final manuscript. Authors (MS, JK, AD) accessed and verified the data.

## Data sharing statement

The data that support the findings of this study are available on request from the corresponding author.

## Declaration of interests

All authors have submitted the ICMJE COI form. Authors (MS, JK, FCW, SC, SE, SB, JG, NB) declare no conflict of interest.

Authors (AD, MJD, PAG, RB, SK, GPA) have declared the following conflicts of interest.

AD: Received grants or contracts from ARMS-Hub study (NIHR PHR) and BOOST trial (NIHR RfPB). Participates in the NIHR BASIS trial Drug Monitoring Committee. MJD: Received grants or contracts from AstraZeneca, Novo Nordisk, Boehringer Ingelheim, Janssen, Sanofi-Aventis, and Eli Lilly. Consulting fees from Lilly, Boehringer Ingelheim, Novo Nordisk, and Sanofi. Honoraria for lectures and presentations from Boehringer Ingelheim, Lilly, Novo Nordisk, Sanofi, and AstraZeneca. Participates in Data Safety Monitoring Boards for Boehringer Ingelheim, Eli Lilly, Novo Nordisk, Sanofi, Carmot, Zealand Pharma, Pfizer, Medtronic, and AstraZeneca. PAG: Received grants or contracts from EPSRC, BBSRC, MRC, Wellcome Leap, and NIHR. Honoraria for lectures from the International Society for Magnetic Resonance in Medicine (ISMRM). Holds leadership roles as Secretary of ISMRM and Councillor for Nottinghamshire County Council and Rushcliffe Borough Council. Received equipment or services from ASG and Philips. RB: Consulting fees from GSK, Novonordisk, and Boehringer Ingelheim. Honoraria for lectures from Gilead and Abbvie. SK: Received grants from UK NIHR for the MHIN Grant Alcohol Assertive Outreach Study, MAHSC Grant for Early Detection in Liver Fibrosis Study, and EME Grant for the MORE-KARE Study. Honoraria for a presentation to the British Association of Psychopharmacology. GPA: Received grants from NIHR, EU DILI consortium, and Gilead. Consulting fees through the Nottingham University Consultants team.
