# Supplementary material for: Association between glucagon-like peptide-1 receptor agonists use and change in alcohol consumption: a systematic review
Source: eClinicalMedicine. 2024 Nov 14;78:102920. doi: 10.1016/j.eclinm.2024.102920 (PMC11701477; doi:10.1016/j.eclinm.2024.102920)
Supplement: Supplementary Tables S1–S3 [file mmc1.docx]

**Supplementary material**

Contents

[S1 Table: Sample search strategy for Ovid Medline, Embase, and PsycINFO 2](#_Toc176533429)

[S2 Table: Individual study inclusion criteria and indication for GLP-1 RA use 4](#_Toc176533430)

[S3 Table: Adverse and serious adverse events reported in studies investigating the role of GLP-1 analogues in alcohol use 5](#_Toc176533431)

# S1 Table: Sample search strategy for Ovid Medline, Embase, and PsycINFO

| **Ovid Medline** | |
| --- | --- |
| 1 | exp Glucagon-Like Peptide 1/ |
| 2 | (glp1 or "glp-1" or glp1r or glp1ra or "glp-1R*" or "glucagon like protein 1" or "glucagon like protein one" or "glucagon like peptide 1" or "glucagonlike peptide 1" or (GLP adj3 (agonist* or analog*))).mp. |
| 3 | exp Glucagon-Like Peptide-1 Receptor/ |
| 4 | (semaglutide or ozempic or wegovy or rybelsus or liraglutide or victoza or saxenda or tirzepatide or mounjaro or zepbound or dulaglutide or trulicity or exenatide or byetta or bydureon or bcise or albiglutide or eperzan or tanzeum or lixisenatide or lyxumia or adlyxin).mp. |
| 5 | 1 or 2 or 3 or 4 |
| 6 | exp Alcohol-Related Disorders/ |
| 7 | (alcoholism or "alcohol use disorder*" or AUD or "high risk drinking behaviour*" or "high risk drinking behavior*" or arld or metALD or "ethanol abuse*" or "alcohol related disorder*" or "alcohol* intox*" or "alcohol* problem*" or "alcohol associated liver disease*" or "alcohol related liver disease*").mp. |
| 8 | (alcohol adj3 (abus* or addict* or intoxicat* or dependen* or misuse or "mis use" or drink*)).ti,ab. |
| 9 | limit 10 to humans |
| 10 | exp clinical trial/ |
| 11 | exp clinical study/ |
| 12 | ((clinical adj2 (trial* or stud*)) or "controlled trial*").ti,ab. |
| 13 | 12 or 13 or 14 |
| 14 | 10 and 15 |
| 15 | 11 or 16 |
| **Ovid Embase** | |
| 1 | exp Glucagon-Like Peptide 1/ |
| 2 | (glp1 or "glp-1" or glp1r or glp1ra or "glp-1R*" or "glucagon like protein 1" or "glucagon like protein one" or "glucagon like peptide 1" or "glucagonlike peptide 1" or (GLP adj3 (agonist* or analog*))).mp. |
| 3 | exp Glucagon-Like Peptide-1 Receptor/ |
| 4 | (semaglutide or ozempic or wegovy or rybelsus or liraglutide or victoza or saxenda or tirzepatide or mounjaro or zepbound or dulaglutide or trulicity or exenatide or byetta or bydureon or bcise or albiglutide or eperzan or tanzeum or lixisenatide or lyxumia or adlyxin).mp. |
| 5 | 1 or 2 or 3 or 4 |
| 6 | exp Alcohol-Related Disorders/ |
| 7 | (alcoholism or "alcohol use disorder*" or AUD or "high risk drinking behaviour*" or "high risk drinking behavior*" or arld or metALD or "ethanol abuse*" or "alcohol related disorder*" or "alcohol* intox*" or "alcohol* problem*" or "alcohol associated liver disease*" or "alcohol related liver disease*").mp. |
| 8 | (alcohol adj3 (abus* or addict* or intoxicat* or dependen* or misuse or "mis use" or drink*)).ti,ab. |
| 9 | 6 or 7 or 8 |
| 10 | exp clinical trial/ |
| 11 | exp clinical study/ |
| 12 | ((clinical adj2 (trial* or stud*)) or "controlled trial*").ti,ab. |
| 13 | 10 or 11 or 12 |
| 14 | exp glucagon like peptide 1 receptor agonist/ |
| 15 | exp alcoholism/ |
| 16 | "incretin mimetic*".ti,ab. |
| 17 | 5 or 14 or 16 |
| 18 | 9 or 15 |
| 19 | 17 and 18 |
| 20 | limit 19 to humans |
| 21 | 13 and 19 |
| 22 | 20 and 21 |
| **Ovid PsycINFO** | |
| 1 | exp Glucagon-Like Peptide 1/ |
| 2 | (glp1 or "glp-1" or glp1r or glp1ra or "glp-1R*" or "glucagon like protein 1" or "glucagon like protein one" or "glucagon like peptide 1" or "glucagonlike peptide 1" or (GLP adj3 (agonist* or analog*))).mp. |
| 3 | exp Glucagon-Like Peptide-1 Receptor/ |
| 4 | (semaglutide or ozempic or wegovy or rybelsus or liraglutide or victoza or saxenda or tirzepatide or mounjaro or zepbound or dulaglutide or trulicity or exenatide or byetta or bydureon or bcise or albiglutide or eperzan or tanzeum or lixisenatide or lyxumia or adlyxin).mp. |
| 5 | 1 or 2 or 3 or 4 |
| 6 | exp Alcohol-Related Disorders/ |
| 7 | (alcoholism or "alcohol use disorder*" or AUD or "high risk drinking behaviour*" or "high risk drinking behavior*" or arld or metALD or "ethanol abuse*" or "alcohol related disorder*" or "alcohol* intox*" or "alcohol* problem*" or "alcohol associated liver disease*" or "alcohol related liver disease*").mp. |
| 8 | (alcohol adj3 (abus* or addict* or intoxicat* or dependen* or misuse or "mis use" or drink*)).ti,ab. |
| 9 | 6 or 7 or 8 |
| 10 | exp clinical trial/ |
| 11 | exp clinical study/ |
| 12 | ((clinical adj2 (trial* or stud*)) or "controlled trial*").ti,ab. |
| 13 | 10 or 11 or 12 |
| 14 | exp glucagon like peptide 1 receptor agonist/ |
| 15 | exp alcoholism/ |
| 16 | "incretin mimetic*".ti,ab. |
| 17 | 5 or 14 or 16 |
| 18 | 9 or 15 |
| 19 | 17 and 18 |
| 20 | limit 19 to humans |
| 21 | 13 and 19 |
| 22 | 20 and 21 |
| 23 | GLP1.ti,ab. |
| 24 | exp "alcohol use disorder"/ |
| 25 | 6 or 7 or 8 or 15 or 24 |
| 26 | 17 and 25 |
| 27 | limit 26 to human |

# S2 Table: Individual study inclusion criteria and indication for GLP-1 RA use

| **Study ID** | **Inclusion criteria** | **Indication of GLP-1 RAs use** |
| --- | --- | --- |
| Klausen et al. (2022) | Eligible patients were aged 18–70, diagnosed with AUD (DSM-5) and alcohol dependence (ICD-10), and seeking treatment. They needed at least 5 heavy drinking days in the past 30 days, defined as consuming 60g of alcohol (men) or 48g (women) per day, as measured by the Timeline Follow Back (TLFB) method | Alcohol use disorder |
| Kalra et al. (2024)^1^ | Adult patients presented to the endocrine clinic and started on liraglutide. | Type 2 Diabetes |
| Probst et al. (2023)^2^ | Individuals aged 18 to 75 who smoked daily and were willing to quit are eligible if they meet at least one of the following criteria: smoke 10 or more cigarettes per day, have moderate cigarette dependence (Fagerström score of 5 or higher), or have a tobacco-related disease. Willing to undergo treatment with varenicline. | Smoking cessation |
| Quddos et al. (2023) | Intervention Group: Participants self-reported taking either Semaglutide (GLP-1 RA) or Tirzepatide (GLP-1 RA/GIP combination) for at least 30 days, active alcohol drinker and BMI ≥ 30. Control: Participants with Type 2 Diabetes and obesity not on GLP-1 RA. | Type 2 Diabetes and obesity |
| Wium-Andersen et al. (2022) | New users of GLP-1 RA and/or DPP-4 agonists, who started treatment after 2009 were included | Type 2 Diabetes |
| Richards et al. (2023) | Ault participants who were treated with semaglutide for obesity and had AUD | Weight loss |
| ^1^Conference abstract, full inclusion criteria not available. Study participants were current alcohol drinkers and alcohol use was measured by using the Michigan Alcohol Screening Tool (MAST). | | |
| ^2^Alcohol consumption was assessed using a standardized questionnaire, asking participants about their average weekly intake of standard glasses of beer, wine, and spirits. In Switzerland, a standard glass is defined as 3 dL of beer, 1 dL of wine, or 0.3 dL of spirits, each containing approximately 10 g of pure ethanol. This method is similar to the timeline followback approach. | | |

# S3 Table: Adverse and serious adverse events reported in studies investigating the role of GLP-1 analogues in alcohol use

|  | **Klausen et al. (2022)** | | **Probst et al. (2023)** | |
| --- | --- | --- | --- | --- |
| **Adverse and serious adverse events** |  |  |  |  |
|  | Exenatide n=62 | Placebo n=65 | Dulaglutide (n=127) | Placebo (n=128) |
| Any serious adverse events | 11 (24) | 8 (19) | 10 (8) | 8 (6) |
| **Gastrointestinal events** |  |  |  |  |
| Nausea | 23 (37) | 10 (15) | 98 (77) | 79 (62) |
| Vomiting | 23 (37) | 10 (15) | 45 (35) | 24 (19) |
| Diarrhoea | 1 (2) | 3 (5) | 39 (32) | 49 (40) |
| Abdominal pain | 0 (0) | 1 (2) | 73 (60) | 60 (49) |
| Constipation/change in stool pattern | 3 (5) | 5 (8) | 49 (40) | 35 (28) |
| Reflux | 3 (5) | 2 (3) |  |  |
| Gastroenteritis | 3 (5) | 3 (5) |  |  |
| Gastrointestinal bleeding |  |  | 1 (1) | 2 (2) |
| Acute appendicitis | 1 (2) | 0 (0) |  |  |
| **Infections** |  |  |  |  |
| Respiratory tract infection | 8 (13) | 9 (14) | 33 (26) | 33 (26) |
| Urinary tract infection | 1 (2) | 0 (0) | 7 (6) | 0 (0) |
| Other infections |  |  |  | 2 (1.6) |
| **Mental health and alcohol** |  |  |  |  |
| Worsening anxiety level | 1 (2) | 0 (0) |  |  |
| Depressive mood |  |  | 5 (4) | 7 (6) |
| Suicidal behaviour | 0 (0) | 1 (2) |  |  |
| Suicide (7 weeks after the end of participation) | 1 (2) | 0 (0.0) |  |  |
| Hospitalizations due to withdrawal symptoms | 13 (21) | 10 (15) |  |  |
| **Other adverse events** |  |  |  |  |
| Injection site reactions | 26 (42) | 0 (0) | 33 (26) | 31 (25) |
| Headache | 1 (2) | 4 (6) | 17 (13) | 21 (16) |
| Musculoskeletal | 3 (5) | 2 (3) | 1 (1) | 0 (0) |
| Dizziness/Fatigue | 11 (18) | 5 (8) |  |  |
| Miscellaneous | 19 (31) | 19 (29) | 3 (2) | 2 (1.6) |
| Number (%). There was no data available on AE/SAE for the following studies: Kalra et al. (2024), Quddos et al. (2023), Wium-Andersen et al. (2022), Richards et al. (2023). | | | | |
